# Comparing the effectiveness of transdiagnostic treatment with acceptance and commitment therapy on emotional disorders, rumination, and life satisfaction in patients with irritable bowel syndrome: a randomized clinical trial

**DOI:** 10.1186/s12876-024-03142-w

**Published:** 2024-02-06

**Authors:** Homa Shahkaram, Jafar Sarani Yaztappeh, Amir Sadeghi, Amir Sam Kianimoghadam, Samaneh Soltanabadi, Maryam Bakhtiari, Abbas Masjedi Arani

**Affiliations:** 1 Department of Clinical Psychology, School of Medicine, Shahid Beheshti University of Medical Sciences, Tehran, Iran; 2 Gastroenterology and Liver Diseases Research Center, Shahid Beheshti University of Medical Sciences, Tehran, Iran; 3 Department of Clinical Psychology, School of Behavioral Sciences and Mental Health, Iran University of Medical Sciences, Tehran, Iran

**Keywords:** Transdiagnostic treatment, Acceptance and commitment therapy, Anxiety, Depression, Rumination, Life satisfaction, Irritable bowel syndrome

## Abstract

**Objective:**

The present study was conducted to compare the effectiveness of transdiagnostic treatment (UP) with the acceptance and commitment therapy (ACT) on the emotional disorders, rumination, and life satisfaction in the patients with irritable bowel syndrome (IBS).

**Method:**

The present study was a randomized clinical trial with a pre-test and post-test design. Between the winter of 2021 and the end of spring 2022, Taleghani Hospital in Tehran received referrals from the statistical population of IBS patients. Of them, 30 individuals (15 in each group) were chosen by convenience sampling and then randomly allocated to groups. UP (It is emotion-based and intervenes in comorbid symptoms), and ACT treatments were provided to the participants online. The participants in the UP and ACT groups received the desired treatments in eight weekly sessions of 45–60 min.

**Results:**

There was no significant difference between UP pre-test and ACT regarding depression, anxiety, rumination, and life satisfaction (*P* > 0.05). There was no significant difference between UP and ACT post-test in terms of depression, rumination, and life satisfaction (*P* > 0.05), but due to anxiety, their difference was significant (*P* < 0.05). Besides, there was a significant difference between pre-test and post-test phases of UP and ACT regarding depression, anxiety, and rumination (*P* < 0.05). Still, they had no significant difference regarding life satisfaction (*P* > 0.05).

**Conclusion:**

Therefore, it is suggested that specialists use UP and ACT as effective psychological treatments for the emotional symptoms of IBS patients to improve psychological symptoms.

## Introduction

Irritable bowel syndrome (IBS) is a functional gastrointestinal tract disorder with symptoms, including abdominal pain accompanied by changes in stool form or frequency [1]. Patients with IBS may also experience distention, bloating, food-induced symptoms, localized discomfort variations, and changes in their stool pattern over time [2]. It has been claimed that 1.1–25% of Iranian populace suffer from IBS [3, 4]. IBS is more common in the people with comorbid psychiatric symptoms and young women than in other people [1]. Nearly 12% of patients seek primary care for IBS-related complaints [5, 6]. Recently, it was shown that IBS patients with mental disorders incur higher costs than IBS patients without mental disorders [7].

The factors, such as new disease onset, disease activity, side effects of drugs, stressful life events, hospitalization, and low socio-economic status are likely to affect the mood of IBS patients [8]. While psychological symptoms are not directly part of IBS, they play a role in the course, control, prognosis, and clinical consequences of this disease, along with severe stress, they lead to disturbances in the quality of life of these patients [9, 10]. There is evidence from prior studies that IBS is associated with anxiety and depression [11]. In addition, the evidence demonstrated that IBS patients differ from healthy individuals in terms of anxiety and depression [12–14]. In this regard, the prevalence of anxiety, and depression symptoms in IBS patients was 39.1% and 28.8%, respectively, and the prevalence of anxiety and depression disorders in these patients was 23% and 23.3%, respectively [11]. One of the methods of responding to depressed and anxious mood is rumination, which is determined by repeated thoughts about the symptoms, causes, and consequences of the disease [15].

Much previous evidence showed that worry-rumination can affect the brain-gut axis. Besides, it was identified as one of the basic factors mediating the simultaneous occurrence of emotional disorders (anxiety and depression) in IBS patients [16]. Rumination is repeated and may cause negative feelings such as melancholy, grief, wrath, and resentment. As a result, it can hinder a person's ability to be productive and engage in meaningful activities [17]. Researchers consider rumination a personal characteristic that occurs when a person thinks too much about the pain associated with the disease [18, 19]. In addition, the rumination exacerbates depression symptoms in patients [20], and there are similar results regarding the effect of rumination on anxiety and anger [21]. Rumination due to IBS disease, and enduring severe pain caused by passive thoughts about psychological signs and symptoms in these patients is likely to lead to decreased problem-solving in them [22]. Furthermore, the psychiatric symptoms and disorders have an inverse relationship with life satisfaction [23].

IBS significantly impacts the life satisfaction of patients because to the atypical pain and discomfort associated with irregular bowel habits. Compared to those without IBS, those with IBS report lower levels of life satisfaction [24]. Thus, life satisfaction is influenced by positive factors, such as support, acceptance of illness, coping with difficult situations, levels of self-care, and availability of health care, and negative factors, such as treatment side effects, disease progression, and feelings of loneliness [25].

In psychotherapy, various cognitive behavioral therapy (CBT) interventions are used to reduce anxiety, improve symptoms related to IBS, and improve individual performance [26]. An essential aspect of effectively treating IBS is establishing a robust patient-physician relationship characterized by active listening, empathy, and the establishment of realistic treatment expectations [2, 5, 27]. Recently, attention was paid to transdiagnostic treatment (UP) in different populations, emphasizing mutual components that cause and perpetuate mental disorders [28]. UP is derived from CBT, and focuses on emotions, targets unpleasant emotions, and the patient is trained in the adaptive emotion regulation strategies [29]. UP emphasizes the adaptive nature and application of emotions, and increases awareness of the role of emotions, cognitions, bodily sensations, and behaviors [30]. Acceptance and commitment therapy (ACT) is another treatment derived from CBT that effectively reduces IBS symptoms [31, 32]. The main goal of ACT is to develop psychological flexibility [33]. The core tenet of acceptance and commitment therapy (ACT) is that patients' primary issue is experiencing avoidance. This encompasses the individual's tendency to avoid facing their own thoughts, feelings, sensations, and experiences [34, 35]. Black et al. (2020) showed that interventions based on CBT and hypnotherapy are the most effective to treat IBS [36]. Ito and Muto (2020) showed that ACT significantly affects depression in IBS patients [37]. Thus, Mohsenabadi et al. (2018) showed that UP significantly affects the emotional regulation of IBS patients [38]. Pashang and Khosh Lahjeh Sedgh (2018) showed that ACT significantly increases the life satisfaction of IBS patients [39].

Therefore, considering the effectiveness of psychological treatments, such as CBT and hypnotherapy, it is necessary to determine the effectiveness of new psychological treatments on the psychological symptoms of IBS patients. Given the prevalence of IBS in recent years, psychological variables have a significant role in the development, management, prognosis, and clinical results of this illness. Paying attention to these patients' mental health is one of the important goals that should be considered. Therefore, regarding UP emphasizes the comorbid symptoms of emotional disorders, and targets them, which leads to the simultaneous improvement of the comorbid symptoms of disorders, it seems necessary to compare its effectiveness with treatments, such as ACT that emphasize the specific symptoms of mental disorders. Therefore, the present study was conducted to compare the effectiveness of UP with ACT on emotional disorders, rumination, and life satisfaction in IBS patients.

### Hypothesis

There is a significant difference between the effectiveness of UP with ACT on emotional disorders, rumination, and life satisfaction in IBS patients.

## Method

### Study design

The present research used a clinical trial method with a pre-test and post-test design.

### Research population

The statistical population was IBS patients referred to Taleghani Hospital in Tehran from winter 2021 to late spring 2022.

### Sampling method and sample size

**Fig. 1:**
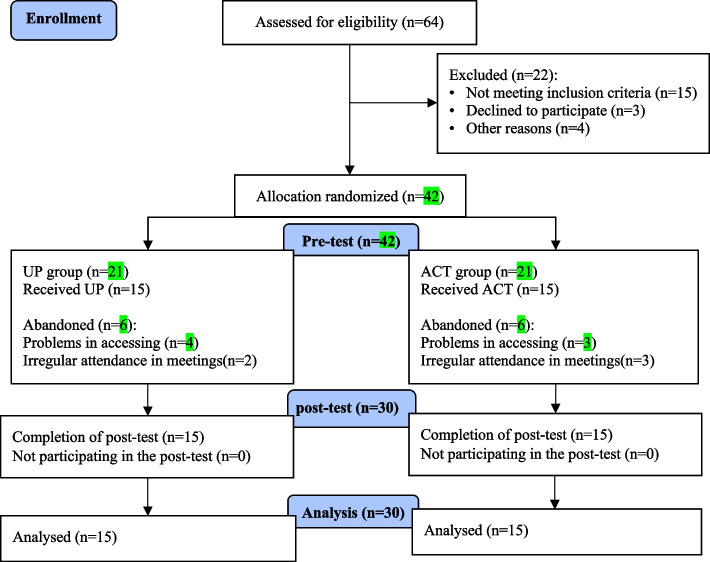
Sampling flowchart in research (*n* = 30)

The present study determined the sample size using Gpower software (effect size = 0.80, alpha = 0.05, and statistical power = 0.80). Therefore, required sample size was 42 (21 people in each group). First, the participants were selected based on the convenience sampling method, and then randomly (based on random allocation law [40]) were assigned to the research groups. The process of registering the participants, and assigning participants to interventions was done by two people from the research team (Ph.D. and Ph.D. clinical psychology students). Six people, however, were eliminated from each group as a result of access issues with certain participants and inconsistent attendance at meetings. In conclusion, the data of thirty people (15 people in each cohort) was analyzed (Fig. 1). One-way blinding was used (participants did not know the type of treatment).

### Inclusion criteria

a) the diagnosis of IBS by a specialist doctor, b) age over 18 years, c) minimum education in reading and writing, and d) written informed consent to participate in the study.

### Exclusion criteria

a) unwillingness to continue treatment, b) incomplete and biased completion of questionnaires, and c) more than two consecutive absences in treatment sessions.

### Procedure

After the research plan was approved, the researcher explained the goals to the IBS patients, and if they agreed to participate in the research, they were provided with a written informed consent form, and pre-test questionnaires to complete. After the sampling process in the pre-test stage, the desired treatments were presented to the participants online based on similar conditions, and specific protocols. Participants in the UP and ACT groups received the desired treatments in eight sessions of 45–60 min weekly. Besides, the participants answered the questionnaires in the post-test phase. Table 1 presents UP protocol from Barlow et al. (2011) [41, 42], and ACT protocol taken from the educational therapy package of Hayes et al. (1999) [34, 43].

**Table 1:** UP protocol adapted from Barlow et al. (2011) and ACT protocol taken from Hayes et al. educational therapy package (1999)

Sessions	Content of the sessions UP	Content of the sessions ACT
**First**	Increasing readiness and motivation to participate in treatment, motivational interviewing, and psychological training for patients' participation and engagement, strengthening self-efficacy, and belief in personal ability, presenting the logic of treatment and determining treatment goals, and providing workbooks, and worksheets for recording the assignments of the sessions	Establishing a therapeutic relationship, introducing the therapist, and explaining the logic of the treatment, providing explanations about the treatment and the effect of mental states on the individual, the family, and its relationship with the symptoms of the disease **Tasks of session:** How did I feel knowing the different feelings and doing the assignment? What did I feel?
**Second**	Psychological training, search and monitoring of emotional experience, recognition of emotions and the concept of learned responses, training of three-component model of emotional experiences, AR model (history, responses, and results), and training of emotional awareness **Behavioral goals:** the awareness of individual emotional response patterns and the factors that create and maintain them **Practicing the learned skills:** monitoring and searching for multiple emotional experiences, determining maintaining factors, such as common triggers, environmental dependencies, and individual emotional response patterns in a sample of emotional experiences	Psychological training, creative frustration, the metaphor of falling into a well **Tasks of session:** An introduction to creative helplessness, an introduction to the practice of experiencing and doing, to gain a better understanding of how the symptoms, thoughts, and feelings associated with IBS interact
**Third**	Learning to observe emotional experiences (emotions and reactions to emotions) using mindfulness techniques, such as non-judgmental and present-focused awareness of emotional experiences **Behavioral goals:** acquiring the skills of objective observation of emotional experiences when they occur and at the moment of doing, identifying thoughts, physical feelings, and behaviors that contribute to discomfort, and understanding the concept of being mindful **Practicing learned skills:** determining how to react, responding to emotions, and practicing non-judgmental, present-focused awareness in multiple emotional experiences	Identifying the client's values and their difference with their goals, clarifying the client's values and obstacles **Tasks of session:** Experiencing and doing, what I have been avoiding? Zorg the alien-Exploring your values, Your 100th birthday
**Fourth**	re-evaluation and cognitive reappraisal, creating awareness of the effect and interrelationship between thoughts, and emotions, identifying self-inconsistent self-evaluations, cognitive reappraisal, and increasing flexibility in thinking **Behavioral goals:** Identifying the role of maladaptive automatic evaluations in creating emotional experiences, identifying thinking patterns, learning ways to correct maladaptive thinking, and flexibility in emotional self-evaluations **Practicing learned skills:** identifying how to react, responding to emotions, non-judgmental awareness, and focusing on the present in the emotional experiences	Examining the client's values and using metaphors related to them, making the right choices in life **Tasks of session:** Identifying values, practice What have I avoided? Values-consistent goals
**Fifth and sixth**	Identifying emotion avoidance patterns and examining behaviors caused by emotion that cause recurrence of disease symptoms Acquaintance and identification of emotion-induced behaviors, and understanding their impact on emotional experiences, identifying maladaptive emotion-demand behaviors (EDBs), and creating alternative action tendencies by confronting emotions **Behavioral goals:** To understand how the emotional patterns of EDBs affect, and their role in the persistence of discomfort and to use them to change the current patterns of emotional responses **Practicing learned skills:** identifying emotional patterns, EDBs, and how it affects the persistence of discomfort in individual emotional experiences	**Fifth:** Psychological training on the concept of defusion, checking the amount of defusion in references, doing exercises for defusion using related metaphors, and teaching to accept internal events **Tasks of session Fifth:** Fusion diary notice and record the moments you engage in one or more forms of fusion, and what it leads to **Sixth:** Psychological education of the concepts of role and context to patients, education of self-observation and self-awareness that does not change **Tasks of session Sixth:** Labeling your experience
**Seventh**	Visceral confrontation and confrontation with situation-based emotions, becoming aware of the logic of emotional dreams, teaching how to prepare a hierarchy of fear and avoidance, designing frequent and effective emotional confrontation exercises visually and objectively, and preventing avoidance **Behavioral goals:** planning the hierarchy of emotional avoidance, including a set of situations and encounters hierarchically, increasing tolerance to emotions and learning new contexts, and using them in new behavioral and emotional experiences **Practicing learned skills:** Endogenous coping exercises aimed at evoking bodily sensations similar to discomfort; these exercises aim to identify the role of bodily sensations in thoughts, behaviors, and their interaction with bodily sensations	Communicating with the present and now, providing training on the effective use of present and now, and training to have a non-judgmental and anticipatory view **Tasks of session:** Looking for the observing self, right here, right now
**Eighth**	Relapse prevention, overview of treatment concepts, and discussion of patient's recovery and treatment progress **Behavioral goals:** using the techniques taught in the previous stages to improve progress to achieve short-term and long-term goals, identifying ways to sustain therapeutic results and possible future problems, practicing practical methods for daily use of therapeutic techniques to improve progress in achieving short-term and long-term goals	Developing effective and developed action patterns, choosing the most valuable behaviors, examining the life story, and summarizing the treatment **Tasks of session:** Turning the acceptance switch on, making a commitment plan, are you living in the bullseye, Building a support team

*UP group* transdiagnostic treatment group, *ACT group* Acceptance and commitment therapy

### Measurement tools

#### Demographic information form

This form was created by the researcher, and included information about the gender, age, marital status, and education of the participants.

#### 21-question depression, anxiety, and stress scale (DASS-21)

This scale is derived from 42-item version and prepared by Lovibond and Lovibond (1995), which has three subscales of depression, anxiety, and stress (each subscale has 7 items). Items are scored on a four-option Likert scale from never = 0 to always = 3. To acquire the final score, multiply the points from this variation by two. According to Lovibond & Lovibond (1995), the correlation between the DASS-21 and the Beck depression and anxiety questionnaires (BDI and BAI) was 0.81 and 0.74, respectively, while Cronbach's alpha for subscales between 0.81–0.91 was noted [44]. Another study reported Cronbach's alpha of this scale as 0.96, and its subscales as 0.87–0.92 [45]. Besides, in Iran, Cronbach's alpha of DASS-21 subscales was between 0.79–0.93, and the total retest reliability and its subscales were reported between 0.74–0.88 [46].

#### Rumination response scale (RRS)

This scale is a subscale of Nolen-Hoeksema and Morrow (1991) Response Styles Questionnaire and has 22 items that are scored on a four-point Likert scale from never = 1 to always = 4. The creators of this scale reported the retest reliability of this scale as 0.67 [47]. Moreover, in another study, Cronbach's alpha of this scale was 0.90, and its retest reliability was reported as 0.67 [48]. Cronbach's alpha coefficient of this scale has been reported as 0.93 in Iran [49].

#### Satisfaction with life questionnaire (SWSL)

This questionnaire was created by Diener, Emmons, Larsen, and Griffin (1985), which has 5 questions and is scored based on a seven-point Likert scale from 1 = completely disagree to 7 = completely agree. They reported the Cronbach's alpha of this questionnaire as 0.87 and its two-month retest reliability as 0.82 [50]. In Bayani et al. research (2016), Cronbach's alpha of this questionnaire was reported as 0.83, and its retest reliability was reported as 0.69 [51].

### Statistical analysis

SPSS-27 statistical software was used for statistical analysis. Frequency, percentage, mean, and standard deviation were used to check the descriptive findings. To ensure that the study groups' demographic characteristics were homogeneous, we used Fisher's exact test for degree of education, an independent t-test for age, and chi-square testing for gender and married status.. To analyze the results related to the research hypotheses, independent t-tests and dependent t-tests (paired) were used. Therefore, all the necessary presuppositions were examined, and all statistical tests were performed at a significance level of 0.95.

## Results

A total of 30 people (15 people in each group) of IBS patients participated. The mean and standard deviation of the age of the participants in the UP group was 33.47 ± 6.68, and in the ACT group, it was 33.67 ± 3.73. Therefore, based on the independent t-test results, there was no significant difference between research groups regarding age (*t* = -0.10, *P* > 0.05). The demographic and clinical information of the participants is shown individually by groups and their homogeneity status in Table 2, including the frequency and percentage.

**Table 2:** The frequency and percentage of demographic and clinical information of the participants separately by groups and their homogeneity status

Variables		UP group (*n* = 15)	ACT group (*n* = 15)	Homogeneity	
		Frequency (%)	Frequency (%)	Statistics	*P*-value
**Gender**	Male	3 (20)	2 (13.33)	0.240^a^	1
	Female	12 (80)	13 (86.67)		
**Marital status**	Single	4 (26.67)	7 (46.67)	1.292^a^	0.450
	Married	11 (73.33)	8 (53.33)		
**Education**	Sub-diploma and diploma	7 (46.66)	2 (13.33)	5.458^b^	0.150
	Associate degree	1 (6.67)	4 (26.67)		
	Bachelor's degree	3 (20)	6 (40)		
	Master's degree	4 (26.67)	3 (20)		
**Depression Pre-Test**	Normal	1 (6.67)	N/A	1.476^b^	0.868
	Mild	N/A	N/A		
	Middle	2 (13.33)	2 (13.33)		
	Severe	4 (26.67)	3 (20)		
	Very severe	8 (53.33)	10 (66.67)		
**Anxiety Pre-Test**	Normal	N/A	N/A	3.128^b^	0.224
	Mild	1 (6.67)	N/A		
	Middle	1 (6.67)	N/A		
	Severe	1 (6.67)	N/A		
	Very severe	12 (80)	15 (100)		
**Rumination Pre-Test**	Mild	N/A	N/A	0.833^a^	0.651

^a ^Chi-square test; ^b^ Fisher's exact test; *UP group* transdiagnostic treatment group, *ACT group* Acceptance and commitment therapy

As shown in Table 2, based on the chi-square test, there was no significant difference between research groups regarding gender, marital status, and rumination pre-test (*P* > 0.05). Furthermore, as determined by Fisher's exact test, there was no statistically significant difference seen across the study groups with regards to education, depression pre-test, and anxiety pre-test (*P* > 0.05). Table 3 shows the mean and standard deviation of the research variables.

**Table 3:** Mean and standard deviation of research variables (*n* = 30)

Variables		Stage	UP group (*n* = 15)		ACT group (*n* = 15)	
			Mean	Standard deviation	Mean	Standard deviation
**Emotional disorders**	Depression	Pre-test	29.47	10.60	28.93	6.88
		Post-test	14.93	5.99	17.33	4.25
	Anxiety	Pre-test	26.80	9.47	29.73	6.63
		Post-test	10.40	5.19	19.47	4.56
**Rumination**		Pre-test	65.93	9.36	67.27	7.42
		Post-test	46.87	8.21	49.40	9.29
**Life satisfaction**		Pre-test	14.53	7.59	16.60	5.38
		Post-test	17.13	8.14	18.13	5.36

*UP group* transdiagnostic treatment group, *ACT group* Acceptance and commitment therapy

In Table 4, the results of the independent t-test for intergroup comparison of the research variables are presented based on the stages of the research.

**Table 4:** Independent t-test results for intergroup comparison of research variables by research stages (*n* = 30)

Variables		Stage	Levene's test		Independent T-test			Confidence interval of 95%	
			*F*	*P*-Value	*t*	df	*P*-Value	Lower	Upper
**Emotional disorders**	Depression	Pre-test	3.95	0.057	0.16	28	0.871	-6.15	7.21
		Post-test	3.08	0.09	-1.26	28	0.216	-6.29	1.49
	Anxiety	Pre-test	0.93	0.344	-0.98	28	0.334	-9.04	3.18
		Post-test	0.09	0.772	-5.08	28	< 0.001^**^	-12.72	-5.41
**Rumination**		Pre-test	1.91	0.178	-0.43	28	0.669	-7.65	4.98
		Post-test	0.57	0.458	-0.79	28	0.435	-9.09	4.02
**Life satisfaction**		Pre-test	2.66	0.114	-0.86	28	0.397	-6.98	2.85
		Post-test	3.85	0.06	-0.40	28	0.694	-6.15	4.15

**P<0.001

As shown in Table 4, based on the independent t-test results, there is no significant difference among the research groups in the pre-test stage regarding research variables (*P* > 0.05). But, as shown in Table 4, based on the results of independent t-test in the post-test stage, there is a significant difference among the research groups regarding anxiety (*P* < 0.001). In Table 5, the dependent (paired) t-test results for the intra-group comparison of the research variables are separately presented for the research groups.

**Table 5:** The results of the dependent (paired) t-test for the intra-group comparison of research variables by research groups (*n* = 30)

Variables		Group	Paired Differences			Confidence interval of 95%		Dependent t-test			Cohen's d
			Mean	Std. Deviation	Std. Error Mean	Lower	Upper	*t*	df	*P*-Value	
**Emotional disorders**	Depression	UP (n = 15)	14.53	11.27	2.91	8.29	20.78	4.99	14	< 0.001^**^	1.29
		ACT (n = 15)	11.60	8.04	2.08	7.15	16.05	5.59	14	< 0.001^**^	1.44
	Anxiety	UP (n = 15)	16.40	9.60	2.48	11.08	21.71	6.62	14	< 0.001^**^	1.71
		ACT (n = 15)	10.27	8.84	2.28	5.37	15.16	4.50	14	< 0.001^**^	1.16
**Rumination**		UP (n = 15)	19.07	12.74	3.29	12.01	26.12	5.80	14	< 0.001^**^	1.50
		ACT (n = 15)	17.87	11.74	3.03	11.36	24.37	5.89	14	< 0.001^**^	1.52
**Life satisfaction**		UP (n = 15)	-2.60	8.24	2.13	-7.17	1.97	-1.92	14	0.242	-0.31
		ACT (n = 15)	-1.53	5.45	1.41	-4.55	1.48	-1.09	14	0.294	-0.28

**P<0.001

*UP group* transdiagnostic treatment group, *ACT group* Acceptance and commitment therapy

As shown in Table 5, there is a significant difference between pre-test and post-test stages of the UP group due to depression (*t* = 4.99), anxiety (*t* = 6.62), and rumination (*t* = 5.80) (*P* < 0.001). Therefore, there is a significant difference between the pre-test and post-test stages of ACT group in terms of depression (*t* = 5.59), anxiety (*t* = 4.50), and rumination (*t* = 5.89) (*P* ≤ 0.001). However, there was no significant difference between pre-test and post-test stages of the UP and ACT groups regarding life satisfaction (*P* > 0.05).

## Discussion

The present study was conducted to compare the effectiveness of UP with ACT on emotional disorders, rumination, and life satisfaction in the IBS patients. In this regard, the between-group analysis results showed no significant difference between research groups in the post-test stage regarding depression. Nevertheless, a notable distinction exists between the two about anxiety. Furthermore, a noteworthy disparity in anxiety and depression levels was observed between the pre-test and post-test phases of the research groups, as indicated by the findings of the within-group analysis. Therefore, the results of the present study show the effectiveness of UP and ACT on emotional disorders. In this regard, Ito and Muto (2020) showed that ACT effectively reduces depressed mood in the IBS patients, but this treatment did not affect the severity of symptoms [37]. Mohsenabadi et al. (2018) showed that UP is effective in the anxiety, and depression of IBS patients which leads to regulating emotions and reducing their symptoms [38]. Azizi and Mohamadi (2016) showed that group dialectical behavior therapy (DBT) is effective in depression in IBS patients and reduces the symptoms of depression in these patients [52]. Therefore, since UP and ACT treatments are similar in nature and main concepts to DBT treatment, they have a similar mechanism of action. In addition, Zemestani and Imani (2016) showed that UP significantly improves the symptoms of depression, anxiety, and emotion regulation in students [53]. In this regard, their results align with the present study's results. Therefore, in explaining the current research results, UP emphasizes the functional nature of emotions, and causes unpleasant emotional reactions to external and internal factors to be extinguished. In addition, cognitive reappraisal abilities are strengthened in UP by addressing issues with negative assessments (overestimating likelihood and catastrophizing), internal and external threatening variables, and developing cognitive flexibility. In UP, using the three-component model of emotion and non-judgmental mindfulness, patients are helped distinguish thoughts, feelings, and behavior from each other correctly. In the moment, they experience them, and the reactions caused by them, and increasing cognitive flexibility, dreaming with unpleasant emotions, and non-judgmental mindfulness lead to the reduction of emotional disorders [29, 41, 42]. Furthermore, with respect to the effects of ACT, it can be stated that this intervention prioritizes committed behavior and values, and that commitment results in the accomplishment of objectives and the attainment of values. This process helps a person get rid of the involvement in negative thoughts and emotions. Another important technique in this treatment is to increase acceptance (thoughts are accepted without trying to control them). Thus, in the defusion technique, a person is taught to consider himself separate from his thoughts and emotions, and observe his thoughts and emotions as an observer [54]. These techniques help a person to significantly manage his emotional symptoms and experience less depression and anxiety as a result. Nevertheless, it is common for anxiety disorders to manifest in conjunction with other mental disorders; therefore, co-occurring symptoms are emphasized in UP. Consequently, UP had a more substantial impact on the anxiety levels of IBS patients compared to ACT in the current study. Therefore, the results of between-group analysis showed no significant difference among the research groups in the post-test stage in terms of rumination. However, based on the results of within-group analysis, there was a significant difference between the pre-test and post-test stages of research groups due to the rumination. Therefore, the results of the present study show the effectiveness of UP and ACT on rumination in IBS patients. Based on the search conducted in domestic, and foreign scientific databases, there was no study on the effectiveness of UP and ACT treatment on rumination in IBS patients. Sharif Ara et al. (2023) showed that rumination is considerably reduced by ACT in individuals with generalized anxiety disorder [55]. According to Shirazipour (2022), ACT has a considerable impact on ruminating in older persons who are depressed [56]. Nasri et al. (2018) showed that UP significantly affects rumination in the patients with type 2 diabetes [57]. Considering that the sample of above studies differed from the sample of the present study, but their results were consistent with the present study. In this regard, in explaining the result of the current research, UP uses techniques, such as recognition of emotions, the identification of emotional experiences, and cognitive reappraisal [41, 42]. Therefore, in recognizing and identifying emotions, people (in the present study, IBS patients) were taught to identify emotions related to rumination, such as sadness. Hence, if IBS patients experience a large amount of sadness related to rumination, using the cognitive reappraisal technique helps to moderate them, which may lead to a decrease in rumination in IBS patients. On the other hand, one of the important skills in ACT is increasing psychological flexibility [33]. Based on previous evidence, rumination has many harmful consequences (poor problem-solving, reduced motivation, reduced concentration, and disturbed cognition). Furthermore, several research have shown the connection between psychological flexibility and rumination. According to a research, practicing mindfulness techniques might improve flexibility, which in turn can lessen confusion and rumination [58]. Also, another component of ACT is cognitive fusion. It occurs when a person's thoughts regulate his behavior ineffectively, and instead of paying attention to the context of thought, the person pays attention to its content. Therefore, when this process dominates the individual's experience, it leads to the psychological inflexibility [34]. Indeed, UP and ACT emphasize accepting unpleasant internal experiences without trying to avoid them, which is likely to lead to a reduction in threatening rumination. In this regard, both treatments can significantly reduce rumination in IBS patients in terms of facing unpleasant experiences and accepting them.

However, the results of the between-group analysis showed that there is no significant difference among research groups in the post-test stage in terms of life satisfaction. In addition, based on the results of within-group analysis, there was no significant difference between the pre-test and post-test stages of the research groups in terms of life satisfaction. As a consequence, the current study's findings did not demonstrate how well UP and ACT affected IBS patients' life satisfaction over the short term. According to Koohneshin Taromi et al. (2021), ACT and UP both significantly affect nurses' life satisfaction, but UP is more effective than ACT [59]. Also, Pashang and Khosh Lahjeh Sedgh (2019) showed that ACT significantly increases the life satisfaction of IBS patients [39]. Therefore, the results of their research were inconsistent with the results of the current research. In explaining this research result, the patients with chronic pain feel helpless due to the long and difficult process of treating diseases. This issue negatively affects the treatment process and their lives, leading to long-term rejection of the disease and, as a result, a feeling of lack of life satisfaction [60]. However, life satisfaction is a component that is affected by a person's evaluation of himself, his present and future life, and his adaptation to his current life, and is created over time. Thus, it can be inferred that life satisfaction has steadily grown in the UP and ACT groups based on the average shown in Table 3. Thus, long-term assessments are necessary to determine how well psychological interventions affect life satisfaction. Finally, due to the pain of IBS disease, it is expected that the life satisfaction of these patients will decrease, and it will take a long time to recover.

### Limitations

The present study had limitations, which can be mentioned as not having a follow-up stage in terms of the lack of access to the participants and online implementation of the research, lack of random sampling, use of self-report tools, and lack of a control group. Therefore, it is suggested to consider the follow-up stage in future research to evaluate the effect of psychological treatments to determine their long-term effect. Also, the sampling of the current research was not done randomly in the initial stage, so caution should be taken in generalizing the results. Besides, it is suggested to consider a control group (waiting list) in future research to compare the effectiveness of psychological treatments. Another limitation of the current study was that, in each group, six people dropped out, these people were excluded from the study at the beginning of study, and their information was not included in the analysis. Therefore, it is suggested to generalize the results with caution.

## Conclusion

This study aimed to compare the effectiveness of UP with ACT on emotional disorders, rumination, and life satisfaction in IBS patients. The findings indicated that there was no discernible change in life satisfaction, rumination, or sadness between the ACT and UP post-test stages. Still, there was a noticeable anxiety difference among them. Therefore, there was a significant difference between pre-test and post-test phases of UP and ACT regarding depression, anxiety, and rumination. Still, there was no significant difference among them regarding life satisfaction. Therefore, it is suggested that specialists use UP and ACT as effective psychological treatments for the emotional symptoms of IBS patients to improve psychological symptoms.

## Data Availability

It is possible to access the data after coordination with corresponding author by email.
